# Revenue for Conducting the Work of the Boards of Dental Examiners

**Published:** 1901-07

**Authors:** V. E. Turner

**Affiliations:** Raleigh, N. C.


### REVENUE FOR CONDUCTING THE WORK OF THE BOARDS OF DENTAL EXAMINERS.[1]

BY DR. V. E. TURNER, RALEIGH, N. C.

### (A) BY TAXATION OF THE PEOPLE.

ALTHOUGH one of the main objects of examining persons wishing to commence practice is to secure protection for the people from inferior dental service, it would seem for such immunity the people should be taxed as they are for any other protection which the law affords. This is doubtless the correct principle. As the members of the State boards are State officers, it would seem most rational that they should be paid as other State officers.

But there would be great difficulty in influencing such legislation, as any proposed enactment relating to dental surgery or any branch of medicine which carried with it a draft upon the treasury of a State would excite the opposition of the average legislator. It would not be expedient to ask this, as it would be fatal to any further amendment to our present dental laws. In fact, in some of the States it is specially provided that under no circumstances shall these dental enactments be of any expense to the State.

### (B) BY FEES FROM THE EXAMINATION OF CANDIDATES.

All things considered, the most satisfactory plan of securing compensation for the service rendered by members of the State examining boards should be derived from the fees received from the applicants for license. All legislation prescribing and controlling the standard of qualifications of licentiates in dental surgery has been effected through the influence of State dental societies, and every one will concede that such enactments are of great benefit not only to the profession, but to all reputable practitioners, in maintaining a higher standard of professional attainments, which gives more dignity and a higher consideration among the other professions as well as the public.

The amount of compensation should be to some extent commensurate with a reasonable expectation of income to be derived

---

[1] Abstract of paper read before the American Medical Association, Section on Stomatology, St. Paul, Minn., June 4, 5, 6, and 7, 1901.

from these fees. Of course, in some States it would be larger than in others, but a per diem of ten or fifteen dollars, and three cents mileage, would not be extravagant remuneration for members of examining boards, except the secretary, who should be paid twenty-five dollars per annum additional.

If these and such other legitimate expenses should aggregate less than the amount of fees received, the balance should be turned over to the State society for conducting scientific research or other educational purposes. If these amounts should aggregate more than the fees, then the State society should supply the deficit.

In addition to these duties, each member must prepare suitable question lists before the meeting, and examine the replies after, in order to give the proper grading to each applicant examined, and both of these require the exercise of much thought and judgment.

(c) BY TAXATION OF THE PROFESSION.

If this should be necessary, then the profession should not hesitate to bear that burden for the sake of the benefit which is derived from a proper discrimination between the worthy and qualified and the unworthy and incompetent, and in order to prevent the lowering of the standards of professional equipment and to provide against the advent of those who pursue the calling as a hustling business rather than a learned and dignified profession.

---

